# Genetics of Bacteria: a call for papers

**DOI:** 10.1093/g3journal/jkae133

**Published:** 2024-06-21

The journals of the Genetics Society of America, GENETICS and G3: Genes|Genomes|Genetics, are calling for submissions of papers on the Genetics of Bacteria.

Classical genetic analyses of bacteria have made numerous and profound contributions to both fundamental and applied science. From early studies on gene regulation and the nature of horizontal gene transfer, through to the recent discovery and harnessing of CRISPR, bacterial genetics continues to yield important advances. Genetic and molecular studies on DNA replication, transcription, genome stability, and mutagenesis have provided insights into essential cellular and evolutionary processes.

This series will highlight recent genetic and genomic work that leverages both classical and innovative genetic approaches in the study of bacterial genetics.

Specifically, we invite high-quality submissions on the genetic diversity of bacteria; genome preservation and chromosomal integrity; mutagenesis and mutational mechanisms; characterization of gene function and regulation; mobile genetic elements and their interaction with the bacterial host; bacteria–phage interaction and coevolution; bacterial defense systems; the genetic basis and evolutionary consequences of bacterial interactions including interference competition or public good production; genetic interactions; the genetic basis of medically important traits like virulence and drug resistance; and theoretical treatments of microbial population genetics.

**Series Editors:**

- Dave Baltrus (University of Arizona, United States of America)
- Houra Merrikh (Vanderbilt University, United States of America)
- Hildegard Uecker (Max Planck Institute for Evolutionary Biology, Germany)
- Alex Wong (Carleton University, Canada)

The series will be launched in a mid-2025 issue of both GENETICS and G3, although articles will be published online shortly after acceptance via Advance Access. Papers will be collected and navigable across the two journals and promoted on social media and other venues in a manner similar to other series the journals have published.

Authors are invited to submit manuscripts by December 2 to their journal of choice. Manuscripts will be reviewed and edited through the standard peer review process and according to the usual high standards of the journals. Some manuscripts submitted to GENETICS may be offered a transfer to G3: Genes|Genomes|Genetics instead.

We encourage continued submissions past 2024 December 2 as we intend this series to be an ongoing and growing resource in this area. At submission, please choose the “Genetics of Bacteria” article type in the submission system and mention the series in your cover letter.

Please share with your colleagues, and do not hesitate to contact any of the series editors or the GENETICS and G3: Genes|Genomes|Genetics Editorial Office with questions, suggestions, or presubmission inquiries.

