# Correction to: Genetics of Bacteria: a call for papers

**DOI:** 10.1093/g3journal/jkae192

**Published:** 2024-08-20

This is a correction to: Genetics of Bacteria: a call for papers, *G3 Genes|Genomes|Genetics*, Volume 14, Issue 7, July 2024, jkae133, https://doi.org/10.1093/g3journal/jkae133

This article has been corrected to update the deadline for submissions of papers on the Genetics of Bacteria to December 2, 2024.

